# Combination of Tin and Gold Fillings

**Published:** 1882-09

**Authors:** A. H. Thompson

**Affiliations:** Topeka, Kansas.


### ARTICLE. V.

### *Combination Tin and Gold Fillings.*

BY A. H. THOMPSON, D. D. S., TOPEKA, KANSAS.

A very interesting discussion occurred in the meeting of the Odontological Society of Pennsylvania, November 5th, 1881, (See "*Dental Office and Laboratory*," January, 1882,) upon the advantages to be derived from combining gold and other filling metals, such as tin or amalgam, in proximate fillings, presenting in the buccal teeth.

Dr. Darby had "long noticed that when gold or amalgam came in contact in the same tooth, that there is always a greater discoloration of the amalgam than when the two do not touch." He had "also observed that the edges of the amalgam which came in contact with the tooth, are much warped, and there is less appearance of shrinkage." He was "firmly of the opinion that it injured practice to let gold and amalgam meet in the same tooth." Just what changes are produced in this union he could not state, but there is undoubtedly some change taking place, which is beneficial to the filling."

Dr. S. G. Perry cuts a narrow channel around the margins of his large amalgam fillings, after hardening, and fills it with gold, securing a close joint.

Dr. Corning "believed there was no more immunity from decay when amalgam was used at the cervical edge than when gold was used. There was much still to be learned about amalgam."

Dr. Register had "noticed and carefully noted, that teeth of a certain quality, re-decaying at the cervical walls, when filled with amalgam at this point, when in communication with gold, remain perfect for many years. I had this forced upon my notice through several failures with gold alone, and at last resorted to amalgam, at this point temporarily to bridge over a stated time for an unanticipated European trip. I now, generally, when finding an otherwise perfect good filling breaking down at the cervical portion, cut away the immediate decay and fill with amalgam, in direct contact with the gold. My impression is, this combination of metals forms a galvanic action which decomposes irritating acids, which would otherwise attack the lime salts. A primary battery of acids, attached to a secondary one of metals, will deplete itself in charging the latter, and then will, in part, flow back until drawn off and used by interruption ; and this flow continues back and forth each time, becoming less and less, until the current (for it is a current, and not undefined), ceases, and there is an equilibrium established—and this, to my mind, is what we have in the mouth by the union of the metals."

Dr. Jack said he "confessed to having learned something in reference to repairing gold fillings in difficult situations with amalgam, his previous inclination having been averse to combining two such apparently opposite thing."

Dr. Jas. Truman said that "experience is altogether on the side of patched cases with amalgam, and it should be more resorted to than has been heretofore the case. The use of tin and gold, as recommended by Dr. Abbott, of Berlin, is based on this idea. The failing of cervical margins has led to a change of practice with many. The superiority of tin and gold led Dr. Jenkins, of Dresden, to use it in all approximate surfaces of bicuspid teeth at the cervical margins. The galvanic action confined itself to the metals in combination, resulting in a dark, hard body, resembling amalgam both as to color and destiny but effectually protecting the margin from decay."

There is so much of interest bearing upon our subject, and its elucidation in the above discussion, that it is copied entire, although it may seem a rather lengthy text. But it is only to the advantages of employing tin and gold in combination in the proximate cavities of bicuspids and molars that we wish to consider at present. The chemistry of the subject and the scientific explanation of the theory of the influence of tin as an adjunct of gold in protecting cervical margins, is sufficiently detailed in the discussion quoted. To these we must add the advantageous properties of tin—the peculiar softness and malleability—which makes it the principal one of the soft working filling metals. These qualities allow of its more perfect adaption to the soft, often frail, and always difficult to reach, cervical margins of approximate cavities, than any of the preparations of gold. The peculiar qualities are well known to the older generation of operators in this country, and it was extensively employed with excellent success. The mechanical advantages, as well as the chemical qualities, which had an effect in preventing caries, were well known to the fathers. But its two disadvantages of discoloration and softness which allowed of too much wear in exposed positions, led to its general disuse. To this may be added the inability of the younger generation to work metals on the "soft," non-cohensive principle, after the introduction of cohesive gold, which accelerated the abandonment of tin.

But in the present mode of employing tin, the two objections are obviated; the discoloration is rendered unobjectionable by using it only in concealed positions, and the disposition to wear is prevented by covering it on exposed surfaces with gold. Discoloration renders tin objectionable for the incisors and canine teeth, except, perhaps, in very deep cervical or lingual cavities, but its use in these teeth is generally unsafe, on account of the possibility of staining the tooth substance, even to some distance from the tin. Its use is confined almost exclusively to the buccal teeth, but as these are most vulnerable upon the proximate surfaces, and fillings, in these positions most liable to failure, the discrimination is appropriate.

The tin is used by packing carefully against the groove about the cervical margins, being careful to bind it into the cavity as it is introduced. The extent to which it may be brought down is governed by the weakness of the cavity margins, the remoteness of the tooth from the front, and the possibility of stains. It is protected from the wear of mastication by covering well at the margin and properly sloping the filling with gold. This is necessary to insure durability as well as to better secure the tin. If the marginal walls are weak, the tin can be brought up to them with greater ease, safety, and possibility of adaption, leaving only secure attachments on each side for the gold. If near the front, as in the bicuspids, only gold can be used against the buccal wall, for the tin will be sure to stain the enamel, and its use is also contra-indicated in places where there is a possibility, even remote, of the stain reaching a visible portion of the tooth. The disposition of the oxide to penetrate the dentine and enamel must ever be borne in mind; for although this will have a certain preventive influence, and prevent the return of caries, even this advantage can not be gained by the sacrifice of appearance and beauty.

Cohesive gold filling, more especially the extensive, difficult, exhaustive, costly kinds, upon the proximate surfaces of bicuspids and molars, have a notorious, provoking disposition to fail at the cervical margins. The experience of most members of the profession is that they are the most unsatisfactory of all operations in filling, on account of their most certain failure at this most vulnerable point. We are all most reluctant to examine these margins when diagonizing a dentine, on account of the almost certainty of finding decay, and the difficulty and uncertainty of its repair.

This failure is not due entirely to defective work, for the fillings of the best operators in gold are not entirely exempt from it, as we can all testify from the patients who have fallen into our hands. It is not usually due to defective preparation of the cavity of decay, to improper manipulation of the gold; but it is due (1st) to the softness of the cervical margins which will not allow of cohesive gold being brought up close and water-tight, (2d) to the difficult access to this portion of the cavity, and the necessarily imperfect contact, and (3d) to the chemical and galvanic properties of the gold which cause the development and concentration of acids upon the dentos at that point. Any one of these reasons we consider sufficient to warrant its abandonment for tin, which, undoubtedly, is not open to these objections but is materially and pratically preferable in view of objections made against gold.

The writer's experience and observation of the remarkable and altogether disproportionately large percentage of failures of the cervical borders of gold fillings on the proximate surfaces of the buccal teeth, led him, some years ago, to determine upon a radical change of his methods of treating these cavities, in the medium and worst classes of teeth at least. Study and experiment in the subject led to the adoption of the tin and gold combination filling, with good results. Several years employment of this method, with almost complete success, has established the conviction that it is by far the best and most satisfactory filling for these cavities. In the few cases where it has failed, the cause has been directly traceable to defective preparation of the cavity, or manipulation of the metals. Where this can be detected, of course the correction is plain and easy, and the practical results are thus under control. But in the case of gold the controling influences are different and beyond our power ; the opposing properties of the material, the difficulties of manipulation, are uncontrollable factors of the operation ; and where success or failure is a matter of accident, with the stabilities in favor of the latter, the candid man must seek something more promising and positive. This, we claim, the tin and gold combination gives us.

The probable bearing which the "New Departure" theories have upon this matter, we have not taken the time to consider ; but it is probable that the various electrical properties of the two metals in contact has much to do with the success of the method. The compatibilities are certainly more nearly adjusted. But we cannot go into this branch of our subject, and prefer briefly to place the method before you as a safe and practical thing.—*Proceedings of Kansas State Dental Association.*

